# A Novel Fault Diagnosis Method of Rolling Bearings Based on AFEWT-KDEMI

**DOI:** 10.3390/e20060455

**Published:** 2018-06-11

**Authors:** Mingtao Ge, Jie Wang, Fangfang Zhang, Ke Bai, Xiangyang Ren

**Affiliations:** School of Electrical Engineering, Zhengzhou University, Zhengzhou 50001, China

**Keywords:** empirical wavelet transform, hypothesis test, adaptive filtering, kernel density estimation, mutual information, rolling bearings fault diagnosis

## Abstract

According to the dynamic characteristics of the rolling bearing vibration signal and the distribution characteristics of its noise, a fault identification method based on the adaptive filtering empirical wavelet transform (AFEWT) and kernel density estimation mutual information (KDEMI) classifier is proposed. First, we use AFEWT to extract the feature of the rolling bearing vibration signal. The hypothesis test of the Gaussian distribution is carried out for the sub-modes that are obtained by the twice decomposition of EWT, and Gaussian noise is filtered out according to the test results. In this way, we can overcome the noise interference and avoid the mode selection problem when we extract the feature of the signal. Then we combine the advantages of kernel density estimation (KDE) and mutual information (MI) and put forward a KDEMI classifier. The mutual information of the probability density combining the unknown signal feature vector and the probability density of the known type signal is calculated. The type of the unknown signal is determined via the value of the mutual information, so as to achieve the purpose of fault identification of the rolling bearing. In order to verify the effectiveness of AFEWT in feature extraction, we extract signal features using three methods, AFEWT, EWT, and EMD, and then use the same classifier to identify fault signals. Experimental results show that the fault signal has the highest recognition rate by using AFEWT for feature extraction. At the same time, in order to verify the performance of the AFEWT-KDEMI method, we compare two classical fault signal identification methods, SVM and BP neural network, with the AFEWT-KDEMI method. Through experimental analysis, we found that the AFEWT-KDEMI method is more stable and effective.

## 1. Introduction

Rolling bearing is a very important mechanical part in all kinds of rotating machinery. A slight failure of rolling bearing may affect the stability and safety of the system, and thus cause very serious consequences [1,2]. The vibration signal of rolling bearing contains a lot of system dynamics information [3], such as impact signals, especially when the rolling bearing is malfunctioning. Therefore, it is very effective to diagnose faults of rolling bearing by analyzing the vibration signal of rolling bearing.

The vibration signal of rolling bearings is generally nonlinear, non-stationary [4,5] and non-Gaussian [6,7]. At the same time, because of the complexity of the working environment, it contains a large number of noise signals such as Gaussian noise [8,9]. In view of this, it is necessary to find an analytical method that can effectively analyze the non-stationary nonlinear signals and overcome the Gaussian noise interference. Wavelet transform is a good tool to deal with non-stationary signals, but the wavelet decomposition is limited by the wavelet basis function [10,11,12], and the wavelet decomposition layers and thresholds will affect the noise reduction effect when filtering noise [13,14]. Empirical mode decomposition (EMD) [15] has been widely used in non-stationary signal analysis [16,17,18]. However, EMD is also subjected to some problems, such as mode mixing and endpoint effect [19,20,21]. Empirical wavelet transform (EWT) [21] is a new method to deal with non-stationary signals in recent years. It adaptively divides the spectrum of the signal into several frequency bands based on the spectral characteristics of the signal, and the corresponding time-domain signal of each frequency band is a mode of the original signal. EWT avoids the mode mixing problem that existed in EMD [22,23], and creates conditions for more effective extraction of the characteristics of vibration signals. After the vibration signal of rolling bearing is decomposed by EWT method, the modes are obtained. Two problems will be faced when extracting the characteristics of the modes: (1) how to filter the noise (mainly Gaussian noise) interference, because the noise will affect the accuracy of the feature extraction; (2) which modes are selected as the feature extraction object, because the noise is the main component for some modes, meaning they are unsuitable for feature extraction and need to be discarded. In [24], the researchers empirically selected three modes representing low frequencies as objects for extracting signal features, while discarding the remaining modes representing high frequencies. Such an approach is based on the assumption that the noise is only distributed at high frequencies, or the energy of the noise is extremely low at low frequencies. In addition, the number of selected modes can only be determined based on experience. To solve this problem, an adaptive filtering of EWT (AFEWT) is proposed. With this method, first the mode can be obtained by decomposing the signal with EWT, and then the second EWT decomposition is performed on each mode to obtain the sub-mode. A sub-mode represents more detailed information of the original signal. The hypothesis test of Gaussian distribution for each sub-mode is performed. Based on the probability distribution of sub-modes, the sub-modes that are assumed to be Gaussian distribution are regarded as Gaussian noise and filtered out of the original signal. In this way, Gaussian noise in the entire frequency spectrum of the signal can be identified, rather than simply considering that the noise is only distributed at high frequencies. The proposed method is to identify the Gaussian noise distributed over the entire frequency band based on the statistical properties of the sub-modes obtained by the secondary decomposition of EWT. There is no parameter setting problem in this process, and the filtering process is adaptive. At the same time, the modes obtained from the filtered sub-modes are reconstructed. These modes can be considered to have eliminated noise and their components are mainly useful signals, which can be used as feature extraction objects. So the problem of mode selection can be avoided. AFEWT can extract the characteristics of vibration signals efficiently and accurately, which lays a good foundation for fault recognition of rolling bearings.

**Figure 1:**
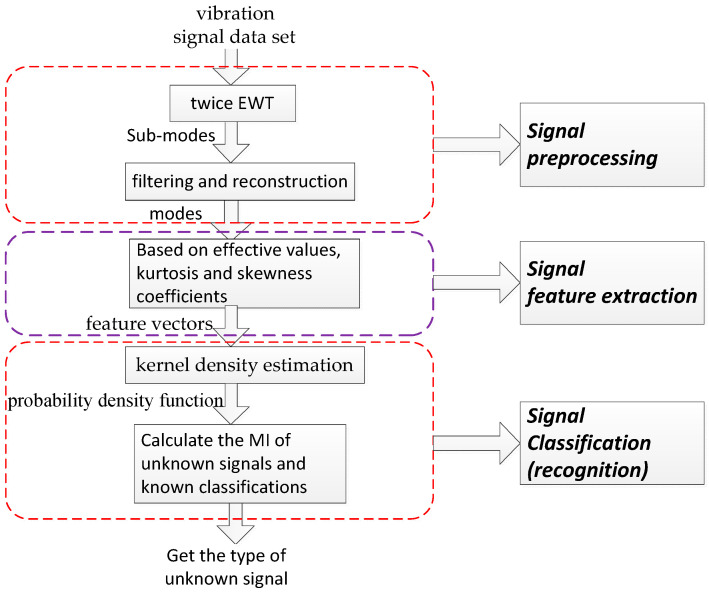
Block diagram of proposed analysis.

It is also essential to construct classifier that recognize normal signals and fault signals. The probability distribution-based Bayesian classifier has a simple design and high execution efficiency, and so it has been widely used in many fields. However, this classifier ignores the dependencies between attributes and assumes the independence of the attributes, which leads to a decrease in the classification accuracy [25,26]. The BP neural network classifier is the most common classifier, but it has slow convergence speed and easily falls into the local minimum, which limits the accuracy of the classification. Compared with the BP neural network classifier, the support vector machine (SVM) classifier has good generalization ability, but the kernel function and kernel function parameters of SVM need to be allocated artificially according to different circumstances, which leads to limited applications [27,28] for SVM. The kernel density estimation (KDE) method describes the distribution characteristics of the data. KDE can obtain many effective characteristics of the data and has no requirements for the prior distribution of the data, so it has been widely used in the engineering field [29,30]. Mutual information (MI) can measure the similarity between two variables from probability distribution well. In this study, a classifier based on the advantages of both KDE and MI is proposed, which is called a KDEMI classifier. First, the probability density of the vibration signal is estimated by KDE. Then, the MI of the unknown signal as well as the probability density of the known signal are calculated. Finally, the classification of the unknown signal is carried out according to the calculation results of mutual information. The block diagram of proposed analysis is shown in Figure 1.

The remainder of this paper is organized as follows: In Section 2, the simulation process is conducted to explain how AFEWT filters Gaussian noise to reconstruct signal modes. In Section 3, a classifier construction method is introduced based on KDEMI. Section 4 introduces a fault diagnosis method based on AFEWT-KDEMI. In Section 5, the validity of the AFEWT method in signal feature extraction and the accuracy and stability of AFEWT-KDEMI method in fault signal recognition are verified by experiments. Finally, conclusions are drawn in Section 6.

## 2. Adaptive Filtering Empirical Wavelet Transform

### 2.1. EWT Principle

Empirical Wavelet Transform (EWT) is an adaptive signal processing method proposed by Gilles et al. in 2013 [21]. This method adaptively divides the signal spectrum into several compactly-supported frequency bands according to the distribution of the local maximum point of the signal spectrum, and each band is subjected to band-pass filter constructed by the wavelet to obtain the corresponding time domain signal. Each time domain signal is a mode component of the original signal. Therefore, the original signal f(t) can be expressed as follows:

(1) $$f(t)=\sum_{k=0}^{N}f_{k}(t),$$

where $f_{k}(t)$ is decomposed component. The method first assumes that the signal spectrum is subdivided into N consecutive parts $\Delta_{n}=[\omega_{n-1},\omega_{n}]$, where $\omega_{n}$ represents the boundary between different parts, and $\cup_{n=1}^{N}\Delta_{n}=[0,\pi ]$. After determining the segmentation interval $\Delta_{n}$, the method defines band-pass filters on each segmentation interval $\Delta_{n}$. Gilles adopted the reconstruction method of Meyer wavelet to reconstruct empirical wavelets. The empirical scaling function ${\hat{\phi }}_{n}(\omega )$ and the empirical wavelet function ${\hat{\psi }}_{n}(\omega )$ can be expressed as follows:

(2) $${\hat{\phi }}_{n}(\omega )=\{\begin{array}{cc} 1, & \left|\omega \right|\leq (1-\gamma )\omega_{n}\\ \cos[\frac{\pi }{2}\beta (\frac{1}{2\gamma \omega_{n}}(\left|\omega \right|-(1-\gamma )\omega_{n}))], & (1-\gamma )\omega_{n}\leq \left|\omega \right|\leq (1+\gamma )\omega_{n}\\ 0, & \mathrm{otherwise} \end{array}$$

(3) $${\hat{\psi }}_{n}(\omega )=\{\begin{array}{cc} 1, & (1+\gamma )\omega_{n}\leq \left|\omega \right|\leq (1-\gamma )\omega_{n+1}\\ \cos[\frac{\pi }{2}\beta (\frac{1}{2\gamma \omega_{n+1}}(\left|\omega \right|-(1-\gamma )\omega_{n+1}))], & (1-\gamma )\omega_{n+1}\leq \left|\omega \right|\leq (1+\gamma )\omega_{n+1}\\ \sin[\frac{\pi }{2}\beta (\frac{1}{2\gamma \omega_{n}}(\left|\omega \right|-(1-\gamma )\omega_{n}))], & (1-\gamma )\omega_{n}\leq \left|\omega \right|\leq (1+\gamma )\omega_{n}\\ 0, & \mathrm{otherwise} \end{array},$$

where:

(4) $$\tau_{n}=\gamma \omega_{n}$$

(5) $$\beta (x)=x^{4}(35-84x+70x^{2}-20x^{3})$$

(6) $$\gamma <\min(\frac{\omega_{n+1}-\omega_{n}}{\omega_{n+1}+\omega_{n}}).$$

Assuming F[.] and $F^{-1}[.]$ are the Fourier transformation and inverse Fourier transformation, respectively. The empirical wavelet high-frequency component is obtained from the inner product of the signal by the empirical wavelet function. The corresponding mathematical expression is as follows:

(7) $$\begin{array}{l} W_{f}^{e}(n,t)=<f(t),\psi_{n}(t)>=\int f(\tau )\bar{\psi_{n}(\tau -t)}d\tau \\ =F^{-1}[f(\omega )\hat{\psi }(\omega )] \end{array},$$

where $W_{f}^{e}(n,t)$ is high-frequency component of the empirical wavelet. Then, the low-frequency component can also be obtained from the inner product of the signal by the empirical scale function:

(8) $$\begin{array}{l} W_{f}^{e}(0,t)=<f(t),\phi_{1}(t)>=\int f(\tau )\bar{\phi_{1}(\tau -t)}d\tau \\ =F^{-1}[f(\omega ){\hat{\phi }}_{1}(\omega )] \end{array},$$

where $W_{f}^{e}(0,t)$ is low-frequency component of the empirical wavelet. Finally, the reconstructed original signal is obtained from the sum of high-frequency and low-frequency components:

(9) $$\begin{array}{l} f(t)=W_{f}^{e}(0,t)*\phi_{1}(t)+\sum_{n=1}^{N}W_{f}^{e}(n,t)*\psi_{n}(t)\\ =F^{-1}[{\hat{W}}_{f}^{e}(0,\omega ){\hat{\phi }}_{1}(t)+\sum_{n=1}^{N}{\hat{W}}_{f}^{e}(n,\omega )*{\hat{\psi }}_{n}(\omega )] \end{array},$$

where $W_{f}^{e}(0,w)$ and $W_{f}^{e}(n,w)$ are the Fourier transformation of $W_{f}^{e}(0,t)$ and $W_{f}^{e}(n,t)$, respectively. Thus, the mathematical expressions of the frequency-modulated, amplitude-modulated signal are as follows:

(10) $$f_{0}(t)=W_{f}^{e}(0,t)\times \phi_{1}(t)$$

(11) $$f_{k}(t)=W_{f}^{e}(k,t)\times \psi_{k}(t).$$

Through the above steps, the complex signal can be decomposed with the modal component of the local instantaneous information. It is more effective and more accurate to extract the signal features from the modes.

### 2.2. The Basic Steps of AFEWT

After the signal is decomposed by the EWT, the information contained in the obtained modes are still complex, so the second EWT decomposition is performed on each mode to obtain the sub-modes. The frequency of signal EWT decomposition is determined by the composition of the signal component. If the frequency of decomposition is small, the information contained in the decomposed mode is still complex, which is not conducive to the hypothesis test. If the frequency of decomposition is large, too much computation will be caused. According to experimental experience, the sub-mode information obtained after decomposing twice is more suitable. By analyzing the statistical characteristics of sub-modes, we can figure out whether each sub-mode satisfies the Gaussian distribution. The sub-modes that are considered to obey Gaussian distribution are regarded as Gaussian noise filtering. In this way, we can get rid of the limitation that traditional filtering methods can only filter out the noise of fixed frequency band. The specific steps are as follows:

(1) EWT decomposition of the signal is performed to obtain mode components;
(2) The second EWT decomposition is conducted for each mode to obtain sub-modes;
(3) Hypothesis test of Gaussian distribution with 95% confidence interval is conducted for each sub-mode. The sub-modes do not satisfy Gaussian distribution, which means that useful signals are dominant and need to be preserved. Otherwise, the sub-modes are regarded as Gaussian noise and should be filtered out;
(4) The mode is constructed based on the result of step (3), and then the signal can be reconstructed.

### 2.3. Simulation of AFEWT 

The simulation signal was adopted to verify the effectiveness of the algorithm. Assume there is a simulation signal y(t)=f(t)+αn(t), where f(t) is a signal that is not contaminated, n(t) is a noise signal that is composed of Gaussian signals mixed by mean and variance of (0, 1), (2, 5) and (4, 10), α is the weight of the noise to adjust the SNR, y(t) is a signal that is contaminated by noise. The mathematical expression of f(t) is as follows:

(12) $$\left\{ \begin{array}{l} f_{1}(t)=(1+0.3\cos(10\pi t))\sin(20\pi t+\sin(15\pi t))\\ f_{2}(t)=\cos(60\pi t+\sin(15\pi t))\\ f_{3}(t)=\cos(460\pi t+\sin(70\pi t))\\ f(t)=f_{1}(t)+f_{2}(t)+f_{3}(t) \end{array}\right..$$

**Figure 2:**
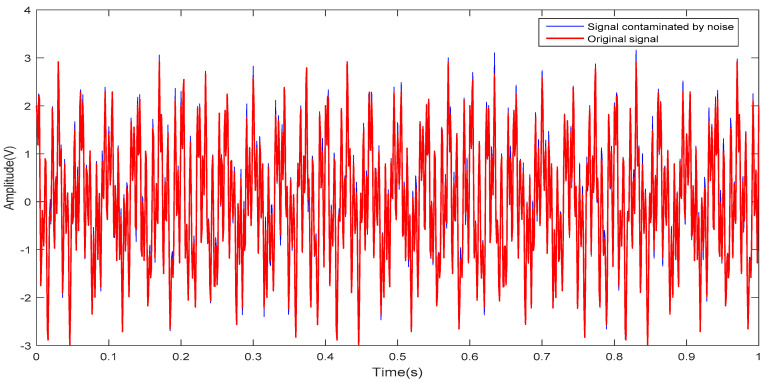
Signal simulation.

**Figure 3:**
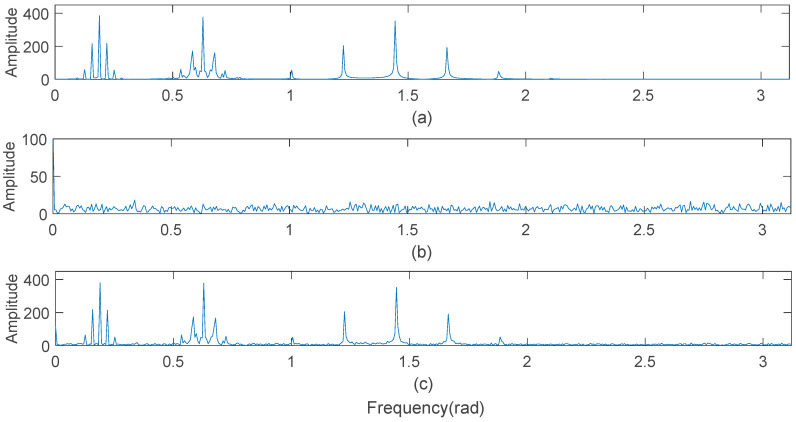
Frequency of the signals: (**a**) original signal; (**b**) noise; (**c**) contaminated signal.

Sampling is performed at a frequency of 1000 Hz in $\left[\begin{array}{cc} 0 & 1 \end{array}\right]$, when the noise weight takes different values, signals with different SNRs can be obtained. When α is 0.01, the *SNR1* is 13.5392. The spectrum of the signal is normalized to $\left[\begin{array}{cc} 0 & \pi \end{array}\right]$. The signal simulation results are shown in Figure 2. The spectrum of the three signals, $f_{}(t)$, αn(t), and y(t), are shown in Figure 3. It is easy to see that the frequency of the noise signal is distributed throughout the frequency band.

**Figure 4:**
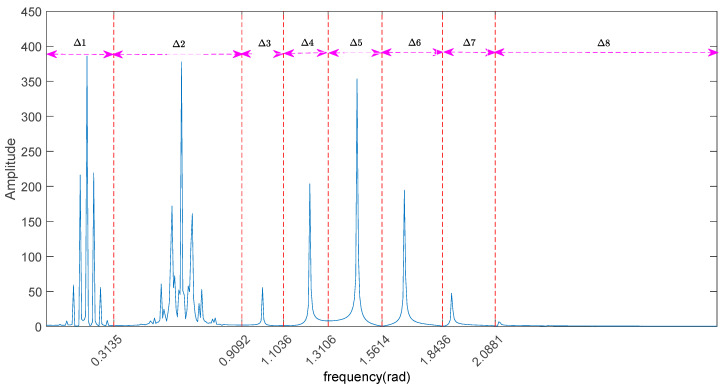
The spectrum of original signal divided by EWT.

**Figure 5:**
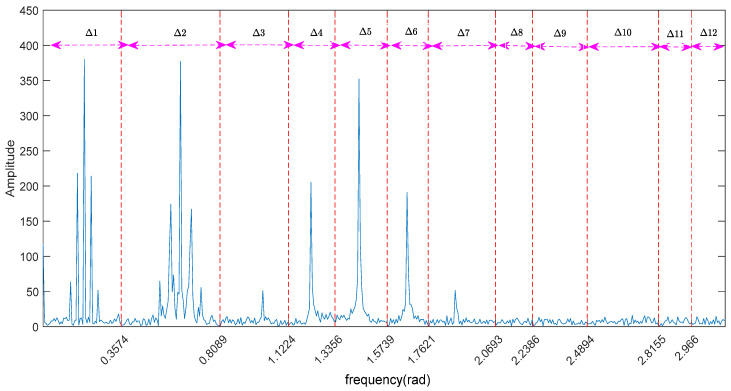
The spectrum of contaminated signal divided by EWT.

Through the Fourier transform of the signal $f_{}(t)$ without noise pollution, the frequency spectrum is obtained. According to the pole characteristic of the frequency spectrum, the EWT adaptively divides the frequency spectrum into eight continuous frequency bands. The frequency bands from low to high are Δ1,Δ2,…Δ8, as shown in Figure 4. The inverse Fourier transform of each frequency band corresponds to one mode of f(t), which are set to $F_{1},F_{2},\ldots F_{8}$, respectively. Similarly, the contaminated signal y(t) is decomposed by EWT. The spectrum is divided into 12 continuous bands Δ1,Δ2,…Δ12, as shown in Figure 5, that is, y(t) has 12 modes.

By comparing Figure 4 and Figure 5, we come to the following the conclusions: (1) Whether there is noise or not, the frequency bands containing the main information of the signal can be found accurately by EWT; (2) Because of the addition of noise, there are more fluctuations in the spectrum of signal y(t), and the fluctuation is observed to distributed all over the $\left[\begin{array}{cc} 0 & \pi \end{array}\right]$ spectrum, that is, the noise exists in high and low frequency; (3) The spectrum of signal y(t) is more divided into four frequency bands than the spectrum of signal $f_{}(t)$ in the high-frequency band.

The second EWT decomposition is performed on each mode of y(t) to obtain the sub-modes. For example, the EWT decomposition of the *F1* mode will lead to four sub-modes. The sub-mode reflects the more detailed information of the original signal. The Gaussian distribution hypothesis test is carried out for each sub-mode with a confidence level of 95%. The results of the test are shown in Table 1.

Here, “1” means that the sub-mode satisfies the hypothesis of “not obeying Gaussian distribution”, which needs to be retained. On the contrary, “0” represents noise needs to be filtered out. It was worth noting that the *F11* mode, whose sub-modes are all considered to be Gaussian noise, should be filtered out. After filtering out the sub-modes identified as noise, the modes were reconstructed and the signals were reconstructed from each mode.

The value of noise weight α was adjusted to 0.08 and 0.2, respectively, and the mixed signal of *SNR2* = 1.4980 and *SNR3* = −6.4608 were obtained respectively. In the case of three SNRs, the comparative analysis of filtering effect among AFEWT, the traditional median filtering, moving average filtering and wavelet filtering method was carried out. The results are shown in Table 2.

The median filter, moving average filter, and wavelet filter are all subjected to the problem of parameter selection. The filter results in the above table are the optimal results obtained by weaving through the possible parameter space. The simulation experiments showed that the AFEWT method not only effectively filtered out Gaussian noise in different frequency bands, but also solved the problem of mode selection in the signal feature extraction. Through simulation analysis, we can know that noise and useful signals can be both high and low frequency. AFEWT is based entirely on the distribution of the signal itself, so the problem of parameter selection will not occur in the filtering process, and this method is well adaptive. After filtering by AFEWT, the low-frequency and high-frequency noise was filtered out, and the low-frequency and high-frequency useful signals were preserved. Therefore, the modes reconstructed by the sub-modes can be used as feature extraction objects. How to select the modality is not a problem in [24].

## 3. KDEMI Classifier

### 3.1. Basic Principles of Kernel Density Estimation and Mutual Information

The kernel density estimation (KDE) is a method to study the data distribution characteristics and estimate the density function of the unknown dataset, without the requirement of any prior knowledge of data distribution or any assumptions to the data distribution. 

The Gaussian kernel is usually used as the kernel function for nuclear density estimation. In terms of data $\left\{x_{i},i=1,2,\ldots n\right\}$, the kernel density estimation is defined as:

(13) $${\hat{f}}_{h}(x)=\frac{1}{nh}\sum_{i=1}^{n}k(\frac{x-x_{i}}{h}),$$

where k(•) is kernel function, and h is bandwidth. Bandwidth h is set to 0.15 times of the predicted confidence interval of variable x to prevent excessive deviation and variance. Mutual information (MI) is an information measure that can effectively measure the degree of overlap between the information of two random variables based on the probability distribution. Probability density reflects the probability distribution characteristics of data. There is a difference in the probability distribution of different types of data, and this difference can be measured by the size of mutual information. According to Equation (13), the probability density functions of two random variables *X* and *Y* are $p_{X}(x)$ and $p_{Y}(y)$, respectively, and $p_{XY}(x,y)$ is their joint probability density function. Their mutual information can be expressed as

(14) $$I(X,Y)=\sum_{x,y}p_{XY}(x,y)\log\frac{p_{XY}(x,y)}{p_{X}(x)p_{Y}(y)}.$$

### 3.2. Basic Principle of Classifier

**Figure 6:**
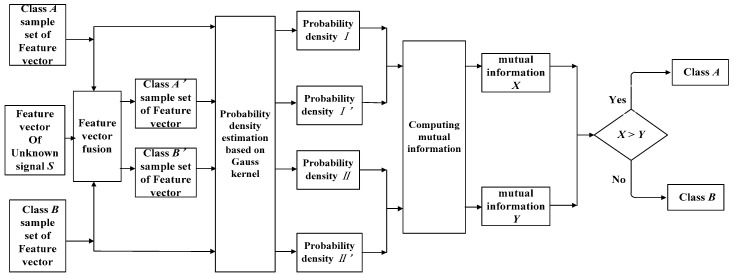
Classification flowchart.

A classifier is able to recognize different types of signals according to their characteristics. In order to extract more detailed and effective features of a certain type of known signal, the signal was first decomposed by EWT to obtain mode components, and then the signal features of each mode component were extracted and the feature vector was constructed through the signal features. A set of feature vectors of the same class of signal was adopted to form sample set *A*. The probability density function *I* was estimated according to Equation (13). Similarly, for the second class of known signals, the feature vector sample set *B* and probability density function *II* were obtained using the same approach. For an unknown class of signal *S*, its feature vector was integrated into the feature vectors sample set *A* and *B*, respectively, resulting in the new sample set *A’* and *B’*. The probability density functions of the feature vectors sample set were recalculated to obtain *I’* and *II’*. The mutual information *X* of *I* and *I’* as well as the mutual information *Y* of *II* and *II’* were calculated. If *X* is greater than *Y*, the signal belongs to the first class of signal; while if *X* is smaller than *Y*, the signal belongs to the second class of signal. The workflow of classification is shown in Figure 6.

## 4. Fault Diagnosis of Rolling Bearing Based on AFEWT-KEDMI

In extracting the characteristics of the rolling bearing vibration signal, the effective value, kurtosis, and skewness coefficient are very effective indicators [6]. The rolling bearing fault identification procedures are as follows:

(1) The vibration signal is decomposed twice with EWT to obtain the sub-modes. Filtering is conducted using AFEWT, and the modes are constructed with the filtered sub-modes;
(2) The effective values, kurtosis and skewness coefficients of each mode are extracted and then integrated into feature vectors;
(3) Multiple groups of the same kind of signals are adopted. The feature vectors are extracted according to step (2), and the feature vector sample set is obtained based on extracted feature vectors.
(4) The Gaussian kernel is used to estimate the probability density of the sample set;
(5) The unknown feature vectors are integrated into the feature vector sample set. The probability density of the new feature vector sample set is recalculated;
(6) After calculating the mutual information of probability density, the purpose of identifying the fault state of the rolling bearing can be achieved according to the category to which the mutual information belongs.

## 5. Experimental Results and Analysis

**Figure 7:**
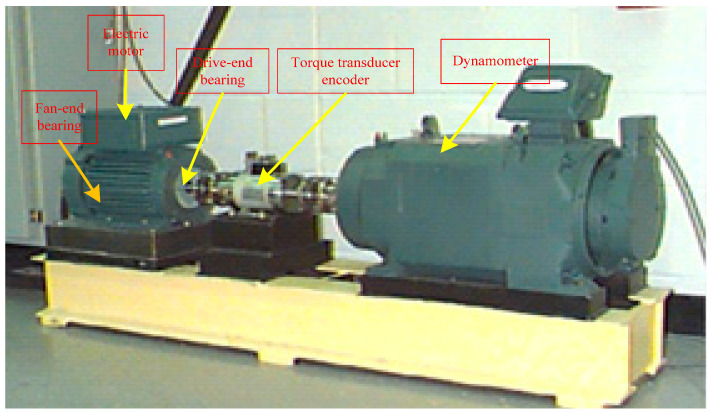
Field test acquisition device.

In this work, data from Case Western Reserve University were used for analysis, and the acoustic emission technique was used for detection. The basic layout of the test rig is shown in Figure 7. It consisted of a 2 HP Reliance electric motor (Cleveland, OH, USA) that drove a shaft on which a torque transducer and encoder were mounted. Torque was applied to the shaft via a dynamometer and electronic control system. Further details about the test setup can be found in [31,32]. During the test process, the diameter of faults was from 0.007 to 0.028 in (0.18–0.71 mm) and faults were seeded on the drive-end and fan-end bearings (SKF deep-groove ball bearings: 6205-2RSJEM and 6203-2RSJEM, respectively) of the motor using an electro-discharge machining (EDM) (Cleveland, OH, USA). The faults were also seeded on the rolling elements and on the inner and outer races, and each faulty bearing was reinstalled (separately) on the test rig, which was then run at constant speed with the motor set to 0–3 horsepower (approximate motor speed was 1797–1720 rpm). The relevant bearing details and fault frequencies are presented in Table 1. During each test, acceleration was measured perpendicularly on the housing of the drive-end bearing (DE), and in some tests acceleration was also measured perpendicularly on the fan-end bearing housing (FE) and on the motor supporting base plate (BA). The length of collected data, N, was 3500. The field test acquisition device is shown in Figure 7 and some basic parameters of the device are shown in Table 3.

**Figure 8:**
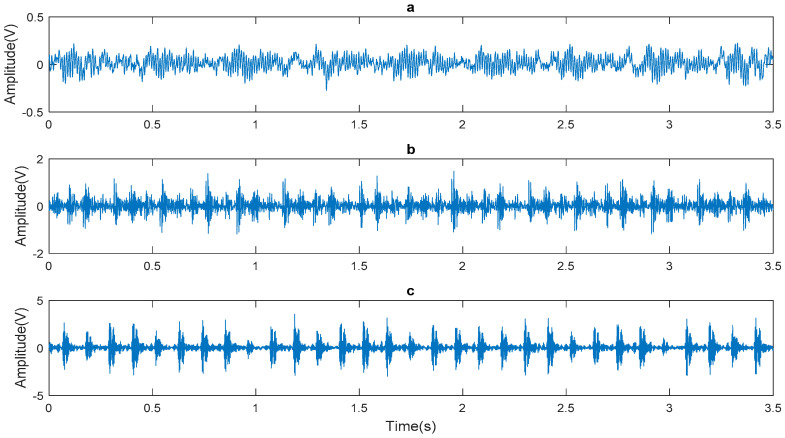
The measured vibration signals: (**a**) normal signal; (**b**) outer race fault signal; (**c**) inner race fault signal.

**Figure 9:**
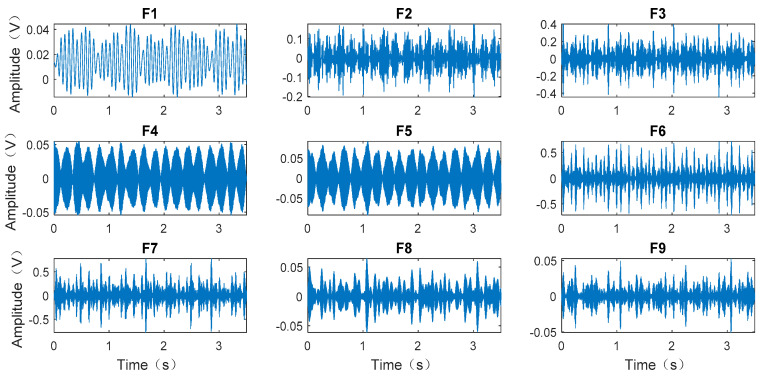
The inner ring fault signal decomposed by EWT.

The collected normal signal, inner ring fault signal, and outer ring fault signal are shown in Figure 8. This study mainly analyzes the inner ring fault signal due to limitation of article length. The inner ring fault signal is subjected to EWT decomposition and the results are shown in Figure 9.

As shown in Figure 8, the inner ring fault signal was decomposed by EWT to obtain nine mode components, denoted as *F1*, *F2*, ..., *F9*, respectively. The mode components reflected the characteristics of frequency components in the signal.

**Figure 10:**
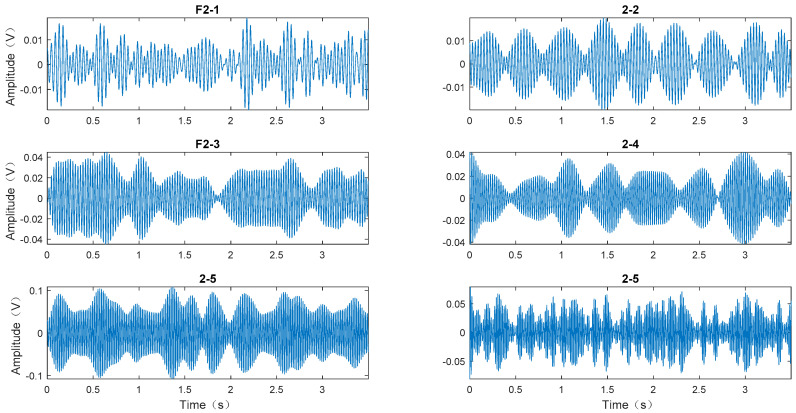
F2 mode decomposed by EWT.

A secondary EWT decomposition was performed for each mode to obtain the sub-modes. Taking *F2* as an example, it was decomposed twice to obtain six sub-modes, denoted as *F2-1*, *F2-2*, ..., *F2-6*, as shown in Figure 10. For the six sub-modes of *F2*, a hypothesis test of the Gaussian distribution was conducted and the result was [0 1 1 1 1 1]. That indicated that *F2-1* obeyed Gaussian distribution noise. So when the mode *F2* was rebuilt, we had to go to *F2-1*. Each mode was reconstructed according to the way of *F2* reconstruction.

**Figure 11:**
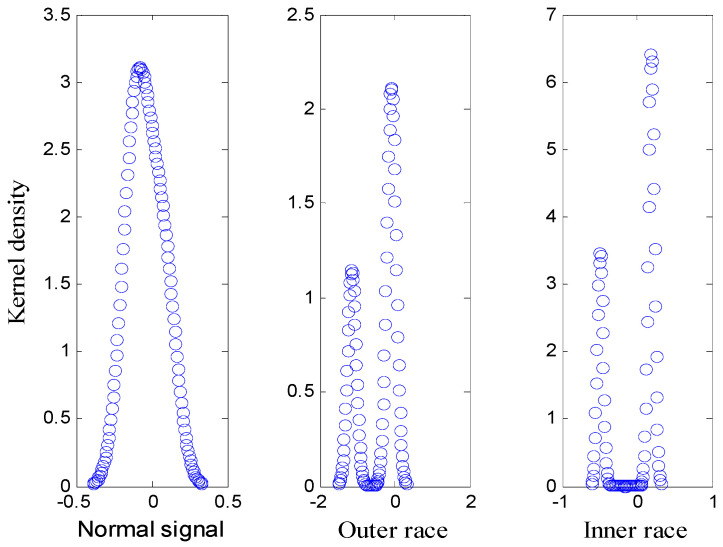
Probability density based on KDE.

Based on 30 sets of normal signals, outer ring fault signals, and inner ring fault signals, AFEWT filtering was performed and the modes of the signals were constructed. The effective value, kurtosis, and skewness coefficient were extracted to form a feature vector, and the probability density of each type of signal was calculated. The result is shown in Figure 11. From Figure 11, we can easily see that the probability density function of feature vector of different classes of signals is quite different. This difference lays the foundation for us to do signal recognition.

**Figure 12:**
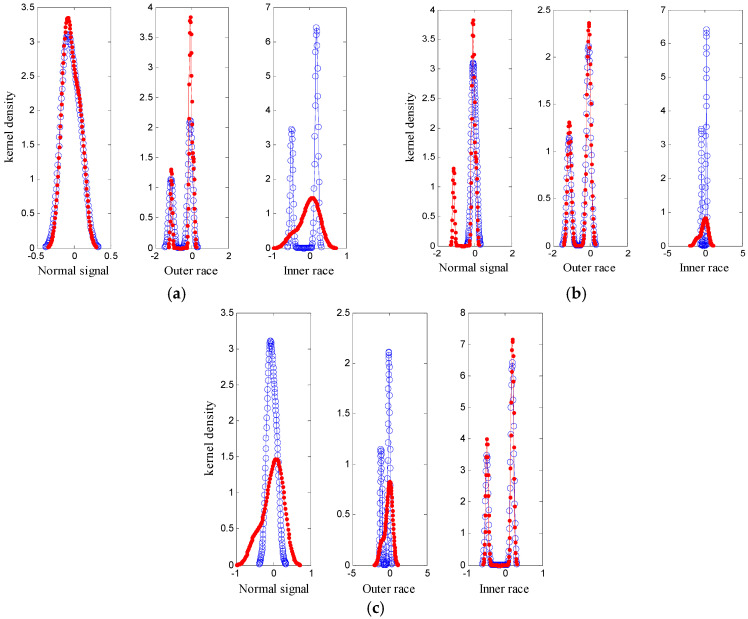
The result of test sample: (**a**) normal signal; (**b**) outer race; (**c**) inner race.

Another 10 sets of signals in three categories were tested. After the feature vector of a given signal was incorporated into the known feature vectors, the probability density was recalculated. The results are shown in Figure 12. As shown in Figure 12, when the added signal and the sample set are of the same class, the probability density coincides with a higher degree, whereas the probability density differs greatly. 

The mutual information between the probability density of the feature vector before adding the test signal and the probability density after adding the test signal was calculated. The results are shown in Table 4. According to the data in Table 4, we know that when the signal and a certain class of signal have high mutual information, it should belong to this kind of signal more.

In order to illustrate the performance of AFEWT method in signal feature extraction, EMD method and EWT method were also employed in signal feature extraction for a comparison. In the experiment, with the same bearing, we used 100 sets of data for training and 30 sets for testing. For comparison, we use different bearing data for training and testing. The training data is 100 groups. The test data is 30 groups, using the same classifier KDEMI. The performances of proposed AFEWT-KDEMI, EWT-KDEMI, and EMD-KDEMI were compared. The results of the test are shown in Table 5.

When extracting signal features, EMD has mode mixing problem and EWT has noise interference and mode selection problem. Fortunately, AFEWT overcomes the problems of the two methods. The experimental results in Table 5 also verify this conclusion very well: AFEWT-KDEMI method can identify all kinds of signals more accurately than EWT-KDEMI and EMD-KDEMI when the classifier is the same. It also shows that AFEWT can extract signal features more effectively.

**Figure 13:**
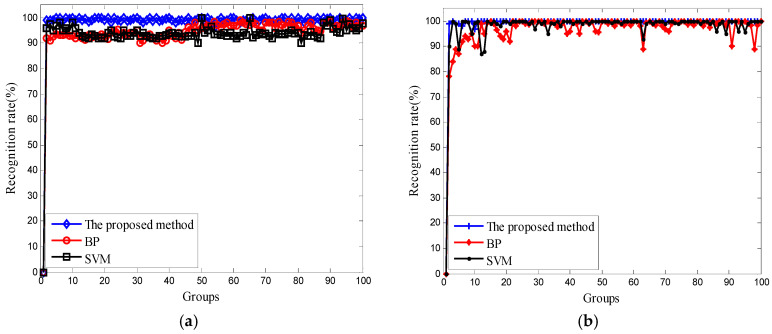

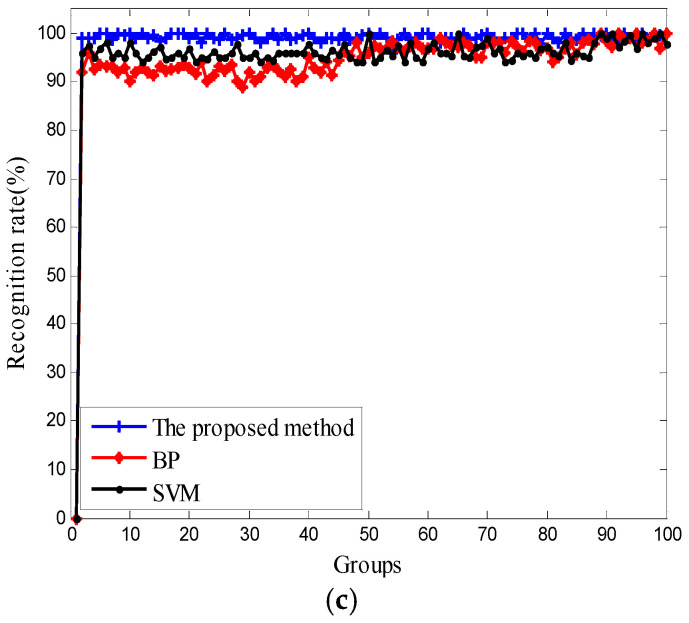
Comparison of the classification accuracy of the three methods: (**a**) normal signal; (**b**) outer race; (**c**) inner race.

In order to test the validity of the proposed method in fault diagnosis, AFEWT-KDEMI method, BP neural network, and SVM, respectively, were used to identify the three signals: normal signal, outer ring fault signal, and inner ring fault signal. The BP neural network has a strong ability of nonlinear mapping and can adaptively store the learning content in the network weight. However, he uses the gradient descent method to train the network. When the error of the weight changes little, the training speed becomes very slow, which affects the speed of convergence. In addition, the BP algorithm is a local search optimization method; therefore, the algorithm is likely to fall into local extremes, causing the training to fail. The generalization performance of SVM is very high because it can separate two kinds of sample data accurately by constructing a decision hyperplane above the two-dimensional plane, so that the samples can be separated to the maximum degree, that is, the separation edge between the two kinds of data points is maximized. However, there are problems of parameter selection and kernel function selection in the practical application of SVM, which limits the applications of SVM. In this experiment, we have adopted the radial basis function (RBF), which has wide applicability. The results are shown in Figure 13. From Figure 13, we can see that no matter what state the rolling bearing is in, the AFEWT-KDEMI method has a higher recognition rate than the other two methods, and the effect is more stable.

## 6. Conclusions

A novel rolling bearing fault diagnosis method called AFEWT-KDEMI is presented in this paper. First, the characteristics of rolling bearing vibration signal are analyzed. The signal has non-stationary and non-Gaussian characteristics, and contains lots of Gaussian noise. In view of the above characteristics, an AFEWT solution is proposed, and experiments show that the scheme can extract signal characteristics very well. Then, combining the advantages of KDE and MI, a KDEMI classifier is proposed. Finally, we conclude through experiments that the AFEWT-KDEMI method proposed in this paper is more accurate and stable than the traditional SVM and BP methods.

## Figures and Tables

**Table 1:** The hypothesis test result of F1 mode.

Mode	Sub-Mode							
	1	2	3	4	5	6	7	8
*F1*	1	1	1	0				
*F2*	0	1	1	1	1	0	1	
*F3*	0	0	1	0				
*F4*	1	0	0	1	0	1		
*F5*	0	1	1	0	0	0	0	
*F6*	1	1	1	1	0	1	1	0
*F7*	0	0	1	0				
*F8*	1	0	1	0	1			
*F9*	0	1	0	0				
*F10*	0	0	0	1	0			
*F11*	0	0	0	0				
*F12*	0	1	0	0	1	0		

**Table 2:** The filtering results with different SNRS.

Original SNR	SNR after Filtering			
	Median Filter	Moving Average Filter	Wavelet Filter	AFEWT
13.5392	9.3115	13.5392	5.9103	14.0713
1.4980	2.6651	3.5997	3.3790	7.7808
−6.4608	−3.4384	−3.2688	−2.5217	−2.4956

**Table 3:** Bearing details and fault frequencies.

Position on Rig	Model Number	Fault Frequencies (Multiple of Shaft Speed, KHz)		
		Outer Race	Inner Race	Rolling Element Ball
Drive end	SKF6205-2RSJEM	23.585	15.415	22.357
Fan end	SKF6203-2RSJEM	21.053	14.947	21.994

**Table 4:** The mutual information of different conditions.

Condition	Mutual Information		
	Normal Signal	Inner Race	Outer Race
Normal signal	0.9117	0.1267	0.1471
Inner ring fault	0.26017	0.9042	0.1815
Outer ring fault	0.1892	0.2741	0.8330

**Table 5:** The accuracy of different fault diagnosis methods.

Different Diagnosis Methods	Training Data	Testing Data	Accuracy (100%)		
			Normal Signal	Outer Race	Inner Race
AFEWT-KDEMI	100	30	96.7	100	96.7
EWT-KDEMI	100	30	93.2	96.7	93.2
EMD-KDEMI	100	30	86.7	90.1	86.7
